# Elements of Practical Medicine

**Published:** 1885-08

**Authors:** 


ELEMENTS OF PRACTICAL MEDICINE. *By* ALFRED H. CARTER, M. D., *London. Member of the Royal College of Physicians, London; Physician to the Queen's Hospital, Birmingham; Emeritus Professor of Physiology, Queen's College, Birmingham; Examiner in Medicine for University of Aberdeen, etc. 12mo.* D. APPLETON & CO., *N. Y. Chicago:* W. T KEENER, *96 Washington St.*

This work, as its title indicates, is an elementary work and is not intended for the practitioner, but for the student as an introduction to the study of medicine. The author has arranged his subjects under their respective sections in a condensed and clear form, robbing them of many points not generally accepted by the medical world, and giving the latest accepted facts. The book will be particularly valuable to the student who is a candidate for hospital appointment, and the therapeutic index may also be a source of comfort to him.

